# Development of neural oscillatory activity in response to speech in children from 4 to 6 years old

**DOI:** 10.1111/desc.12947

**Published:** 2020-03-03

**Authors:** Paula Ríos‐López, Nicola Molinaro, Mathieu Bourguignon, Marie Lallier

**Affiliations:** ^1^ BCBL ‐ Basque Center on Cognition, Brain and Language Donostia/San Sebastian Spain; ^2^ Ikerbasque Basque Foundation for Science Bilbao Spain; ^3^ Laboratoire de Cartographie fonctionnelle du Cerveau Universite libre de Bruxelles Brussels Belgium

**Keywords:** coherence, language development, language lateralization, neural entrainment, reading acquisition, speech perception

## Abstract

Recent neurophysiological theories propose that the cerebral hemispheres collaborate to resolve the complex temporal nature of speech, such that left‐hemisphere (or bilateral) gamma‐band oscillatory activity would specialize in coding information at fast rates (phonemic information), whereas right‐hemisphere delta‐ and theta‐band activity would code for speech's slow temporal components (syllabic and prosodic information). Despite the relevance that neural entrainment to speech might have for reading acquisition and for core speech perception operations such as the perception of intelligible speech, no study had yet explored its development in young children. In the current study, speech‐brain entrainment was recorded via EEG in a cohort of children at three different time points since they were 4–5 to 6–7 years of age. Our results showed that speech‐brain entrainment occurred only at delta frequencies (0.5 Hz) at all testing times. The fact that, from the longitudinal perspective, coherence increased in bilateral temporal electrodes suggests that, contrary to previous hypotheses claiming for an innate right‐hemispheric bias for processing prosodic information, at 7 years of age the low‐frequency components of speech are processed in a bilateral manner. Lastly, delta speech‐brain entrainment in the right hemisphere was related to an indirect measure of intelligibility, providing preliminary evidence that the entrainment phenomenon might support core linguistic operations since early childhood.

## Graphical abstract

Our longitudinal study is the first to reveal that neural phase entrainment to the delta components of speech already occurs at four years of age. Moreover, we show that, at seven years of age, delta speech‐brain entrainment is still bilateral, suggesting that right‐hemispheric specialisation for the perception of speech slow components is not in place.

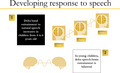

**Research highlights**

- Neural phase entrainment to the slow temporal components of speech already occurs at 4 years of age, and only in the delta‐band frequency range.
- Age‐related increase of coherence to speech in the delta frequency range occurs only in temporal sites of the scalp.
- Delta‐rate entrainment is bilateral at 7 years of age, showing that right‐hemispheric specialization for the perception of speech slow components is not in place.

## 1 INTRODUCTION

In non‐pathological adult populations, the phase of the brain signal at low‐frequency bands (theta band: 4–8 Hz; and delta band: <4 Hz) aligns with the phase of the slow components of the amplitude envelope of speech in a phenomenon known as *speech‐brain entrainment* (Bourguignon et al., 2013; Giraud & Poeppel, 2012; Gross et al., 2013; Peelle & Davis, 2012; Poeppel, 2003). Speech‐brain entrainment has been argued to reflect relevant linguistic operations such as parsing and chunking of the hierarchical linguistic structures of speech (Bourguignon et al., 2013; Ding, Melloni, Zhang, Tian, & Poeppel, 2016; Gross et al., 2013; Meyer & Gumbert, 2018; Meyer, Henry, Gaston, Schmuck, & Friederici, 2016; Molinaro & Lizarazu, 2018; Peelle & Davis, 2012) and the segregation of a target linguistic signal in cocktail‐party situations (Ding, Chatterjee, & Simon, 2014; Ding & Simon, 2012a; Fuglsang, Dau, & Hjortkjær, 2017; Vander Ghinst et al., 2016; Zion Golumbic et al., 2013). Furthermore, abnormal speech‐brain entrainment has been found in children and adults with language disorders such as dyslexia (i.e. developmental reading disorders) which reinforces the idea that appropriate temporal synchronization to speech could be crucial for attaining proper language skills (Di Liberto et al., 2018; Goswami, 2011; Lallier, Molinaro, Lizarazu, Bourguignon, & Carreiras, 2017; Molinaro, Lizarazu, Lallier, Bourguignon, & Carreiras, 2016; Power, Colling, Mead, Barnes, & Goswami, 2016). Theoretical accounts that aim to explain the functional role of entrained oscillatory activity in the brain as the *asymmetric sampling in time* hypothesis (AST; Poeppel, 2003) have suggested that low‐frequency (<8 Hz) phase coherent activity would code for the prosodic and syllabic information of the linguistic signal and show a right hemisphere bias. Indeed, several studies have shown that speech‐brain entrainment at low‐frequency bands is significantly stronger in the right than in the left hemisphere (Bourguignon et al., 2013; Bourguignon, Molinaro, & Wens, 2018; Gross et al., 2013; Molinaro et al., 2016). Note, however, that this assertion applies more accurately to speech delta band components (<4 Hz), whereas results are mixed regarding theta band entrainment (4–7 Hz), with some studies in adults reporting left‐lateralized responses to the syllabic envelope of intelligible speech (Howard & Poeppel, 2012; Peelle, Gross, & Davis, 2013). Meanwhile, according to the AST hypothesis, phonemic information (i.e. information at fast rates) would be preferentially processed in the left hemisphere or bilaterally (Boemio, Fromm, Braun, & Poeppel, 2005; Poeppel, Idsardi, & Van Wassenhove, 2008). Supporting this, there is evidence that gamma band (>30 Hz) oscillatory power synchronization tracking occurs preferentially in the left hemisphere (Boemio et al., 2005; Gross et al., 2013; Poeppel et al., 2008) or bilaterally (Hämäläinen, Rupp, Soltész, Szücs, & Goswami, 2012; Lizarazu et al., 2015). Several research works have suggested that the integration of speech information at fast and slow rates would occur thanks to the orchestration of low‐frequency bands, such that the *slow rhythm(s)* of speech – originated foremost from the amplitude fluctuations of syllables and phrasal prosody units – would be the *acoustic landmarks* upon which fast oscillatory sampling, necessary for the processing of phonemic contrasts, would rely (Doelling, Arnal, Ghitza, & Poeppel, 2014; Ghitza, 2011; Giraud & Poeppel, 2012).

Surprisingly, and despite the remarkable functional relevance that the speech‐brain entrainment phenomenon might have for language acquisition, no study yet has targeted specifically its development during childhood. Nevertheless, the attempts to characterize the neural system supporting speech perception at birth and at different time points during childhood have yielded relevant preliminary findings on which to base predictions on the emergence of speech‐brain entrainment.

### 1.1 Evidence in infants

It is known that human babies process speech in a differential (and preferential) manner as compared to other acoustic signals even before being able to produce speech themselves (Ramus, Hauser, Miller, Morris, & Mehler, 2000; Vouloumanos & Werker, 2007). Nevertheless, the evidence available in infants has brought contradictory results, probably because of differences in the type of responses measured. Whereas studies measuring hemodynamic responses (using fMRI and NIRS) have shown that the new‐born's brain already shows hemispheric specialization for the different spectral and/or temporal features of speech (Dehaene‐Lambertz, Dehaene, & Hertz‐Pannier, 2002; Homae, Watanabe, Nakano, Asakawa, & Taga, 2006; Peña et al., 2003; Perani et al., 2011; Telkemeyer et al., 2009), when oscillatory responses were measured with EEG, either no differences in terms of lateralization (Peña, Pittaluga, & Mehler, 2010; Telkemeyer et al., 2011) or left‐ward asymmetries (Kalashnikova, Peter, Di Liberto, Lalor, & Burnham, 2018) have been found. Regarding hemodynamic responses, infants have been shown to process the prosodic components of speech preferentially in the right hemisphere, both when exposed to natural sentences (Homae et al., 2006; Perani et al., 2011) and to non‐linguistic acoustic signals modulated at different rates (Telkemeyer et al., 2009, 2011). Considering together the (scarce) corpus of NIRS/fMRI evidence from infants and the evidence from adults, one could presume that, in line with the AST proposal (Poeppel et al., 2008), the slowest temporal components of speech (i.e. delta frequency components) are innately processed in the right hemisphere, and no important changes are predicted during language acquisition in childhood. Nevertheless, this conclusion seems hasty for a methodological reason: different techniques were used in the infants as compared to the older children and adults studies. Studies in adults and older children assessing hemispheric specialization for the different temporal components of speech have mostly used measures based on brain phase correlation across trials such as inter‐trial coherence or phase‐locking value via EEG or MEG (Hämäläinen et al., 2012; Lizarazu et al., 2015; Power, Mead, Barnes, & Goswami, 2013) due to their higher temporal resolution as compared to NIRS or fMRI. By contrast, the studies with babies presented above used the changes in the infants' hemodynamic response (Homae et al., 2006; Perani et al., 2011; Telkemeyer et al., 2009, 2011). The comparison among these studies should therefore be performed with caution given the different temporal resolution of the techniques and the different biological processes underlying the responses measured (Nunez & Silberstein, 2000).

Actually, the few studies that have measured oscillatory electrophysiological responses to speech in infants have yielded mixed results. On the one hand, studies that focused their analyses on *power* (amplitude) measures failed to find hemispheric specialization for the different temporal components of speech (Peña et al., 2010; Telkemeyer et al., 2011). On the other hand, the only study that tried to measure entrainment to the speech envelope in infants found larger responses over the left as compared to the right hemisphere when listening both to infant‐ and adult‐directed speech (Kalashnikova et al., 2018). Note, however, that they assessed the infants' Time Response Function (TRF), a measure that might be optimal to pick up evoked components of the auditory response, but not auditory *entrainment* (Doelling, Assaneo, Bevilacqua, Pesaran, & Poeppel, 2019). Overall, as compared to the hemodynamic studies, the few studies measuring oscillatory responses to speech in infants seem to contradict the idea that right‐hemispheric specialization for the temporal processing of the slow temporal components of speech is already in place at birth. At this respect, finding different patterns of lateralization for processing the slow components of speech at different stages of development is not surprising, since it is likely that differential hemispheric responses to different aspects of the linguistic signal interact with the acquisition of linguistic competence (Telkemeyer et al., 2011) and lateralization for different aspects of linguistic processing has been consistently shown to increase with age (Holland et al., 2001; Kadis et al., 2011; Ressel, Wilke, Lutzenberger, & Krägeloh‐Mann, 2008; Spironelli & Angrilli, 2009; Szaflarski, Holland, Schmithorst, & Byars, 2006; cf. Papanicolaou et al., 2006).

### 1.2 Evidence in children

Although analysing oscillatory responses in infants enriches without any doubt the literature on the phenomenon, it is noteworthy that the adults' and the infants' oscillatory responses to speech might be too different to compare. Crucially, speech‐brain entrainment at low frequency bands has been shown to be higher for intelligible versus unintelligible speech (Bourguignon et al., 2013; Gross et al., 2013; Molinaro & Lizarazu, 2018; Peelle et al., 2013). Therefore, while oscillatory responses in babies are most likely due to pure sound analysis, the coherence phenomenon in adulthood seems to be affected by higher order processes such as speech comprehension. Analysing speech‐brain entrainment to the slow components of speech in young children who already understand speech might also bring relevant results for the characterization of its development.

Several studies have reported a consistent right‐lateralized bias of coherence in the theta range using both natural speech (Abrams, Nicol, Zecker, & Kraus, 2008, 2009; Molinaro et al., 2016) and non‐linguistic amplitude‐modulated signals (Lizarazu et al., 2015) in typically developing children. Delta‐band coherence when listening to natural speech has also been shown to be right‐lateralized in children (Molinaro et al., 2016; but see Power et al., 2016). Interestingly, some of these studies also showed that inter‐hemispheric differences in coherence in favour of the right hemisphere were related to reading abilities, such that dyslexic children consistently showed decreased right‐hemispheric coherence at delta or theta frequencies (Abrams, Nicol, Zecker, & Kraus, 2009; Lizarazu et al., 2015; Molinaro et al., 2016). These results suggest that the right‐hemispheric bias for processing the slow temporal components of speech might be related to reading acquisition (Goswami, 2011; Lallier et al., 2018). There is indeed cross‐sectional evidence showing that oscillatory responses change with age, and that these changes could be related to reading experience. Supporting this idea, a cross‐sectional study comparing dyslexic and control children and adults found that coherence to sounds modulated at the phonemic rate (30 Hz) increased significantly with age (Lizarazu et al., 2015). Furthermore, a longitudinal study measuring power synchronization in response to amplitude‐modulated non‐linguistic acoustic signals found that there was an increase in the strength of auditory steady‐state responses in response to 20‐Hz (beta range) amplitude modulations when children were 7 years old (beginning readers) as compared to when they were five (pre‐readers), and that this increase was inversely correlated with reading abilities (De Vos, Vanvooren, Vanderauwera, Ghesquière, & Wouters, 2017). Although this study used power rather than coherence measures, it provides evidence that oscillatory responses to the different temporal components of speech might change with age and be functionally related to reading acquisition.

In any case, existing studies reporting on speech‐brain entrainment in children cannot shed light on its developmental trajectory for several reasons. First and most obvious, all these studies were cross‐sectional, which is not recommended to draw definite developmental conclusions. Second, it is important to remember that oscillatory responses to the low‐frequencies of speech in infants have been found to be bilateral (Peña et al., 2010; Telkemeyer et al., 2011) or predominant over the left hemisphere (Kalashnikova et al., 2018), whereas studies testing older children report a right hemispheric bias (e.g. Abrams, Nicol, Zecker, & Kraus, 2008; Molinaro et al., 2016). This suggests that a shift in laterality is expected during childhood. Importantly, the young participants of the studies measuring coherence were at least 8 years old (Abrams et al., 2008; Lizarazu et al., 2015; Molinaro et al., 2016), and it is well known that speech perception skills are well established at this age. By contrast, no study until now has studied the development of coherence to speech in children who can already pay attention and comprehend speech, but whose speech perception abilities are still developing. Lastly, if right‐lateralized coherence to speech's low frequencies is related to reading skills (e.g. Abrams et al., 2009; Di Liberto et al., 2018; Lizarazu et al., 2015; Molinaro et al., 2016), it could well be that the laterality shift occurs during or after reading acquisition. It is indeed known that the acquisition of reading shapes brain anatomy and function (Carreiras et al., 2009; Dehaene et al., 2010), but since the participants of the children studies mentioned above had acquired reading – more or less successfully – it is not possible to conclude if right‐lateralized coherence to the syllabic and prosodic components of speech is present since early childhood, or if it emerges with literacy.

### 1.3 This study

The main aim of this study was to examine the development of the speech‐brain entrainment phenomenon at low frequencies during early childhood and to characterize its hemispheric specialization. To this goal, we recruited a group of 4‐ to 5‐year‐old children and followed them up until they were 6–7 years old. We used EEG to measure their oscillatory responses to natural speech at three different testing times coinciding with the end of the corresponding school year (t1, second‐to‐last pre‐school year: 4–5 years old; t2, last pre‐school year: 5–6 years old; t3, Grade 1: 6–7 years old). We chose to start testing the children during their fifth year of life for several reasons. First, attention skills of children younger than 4 years are still too immature, which typically leads to excessive movement and discomfort during neurophysiological recordings. Second, according to the American Speech‐Language‐Hearing Association (ASHA; www.asha.org), in the 5th year of life children develop adult‐like grammar and reach an important milestone in their communication skills: the ability to understand most of what they hear and pay attention to short stories. Therefore, before this age we would not have been able to establish clear links between our evidence and the evidence coming from adults and older children, since our intention was to test coherence to *intelligible* speech. Moreover examining children during this period would give us the opportunity to test the hypothesis that reading acquisition affects oscillatory responses to speech, since this cohort of children received formal reading instruction in the period between t2 and t3 (i.e. during Grade 1).

### 1.4 Hypotheses

Since this was the first study analysing speech‐brain entrainment longitudinally in young children, our predictions were based on adult studies analysing coherence measures, and on studies examining the development of speech perception during infanthood and childhood from other fields and/or using different techniques and analyses.

First, if the coherence phenomenon relates to intelligibility (Gross et al., 2013; Molinaro & Lizarazu, 2018; Peelle et al., 2013), and given that during their 5th year of life children are able to *fully* understand speech (with their obvious lexical limitations), we expected coherence to speech to be present since the first testing time (t1) and along the subsequent testing times (t2 and t3).

Concerning developmental effects, we expected coherence to increase with age based on previous cross‐sectional studies using amplitude‐modulated signals at different rates relevant for speech perception (Lizarazu et al., 2015). Our hypothesis was also based on a longitudinal study that tested oscillatory responses in children and adults (age range from 4 to 25 years old) and found that phase‐locking values to musical tones at different frequency bands correlated positively with age (Shahin, Trainor, Roberts, Backer, & Miller, 2010). Lastly, our hypotheses on hemispheric specialization were not definite because whereas studies in babies reported either bilateral or leftward asymmetries for processing the slow temporal components of speech (Kalashnikova et al., 2018; Peña et al., 2010; Telkemeyer et al., 2011), studies in older children (at least 8 years old) measuring coherence have reported a right‐hemispheric bias (e.g. Abrams et al., 2008; Lizarazu et al., 2015; Molinaro et al., 2016). This suggests the existence of lateralization changes that might be functionally related to linguistic experience (Telkemeyer et al., 2009). We hence considered the possibility of finding different lateralization patterns along the children's development, probably related to evolving linguistic skills, and particularly to reading acquisition. Indeed, if hemispheric lateralization is related to reading experience and/or skills (e.g. De Vos et al., 2017; Lizarazu et al., 2015; Molinaro et al., 2016), we expected such switch to occur between t2 and t3 in our study, period during which the children received formal reading instruction for the first time.

## 2 MATERIALS AND METHODS

### 2.1 Participants

The children who participated in this study were taking part in a larger longitudinal study carried out at BCBL. They were recruited from two different public schools in Donostia‐San Sebastián (Basque Country, Spain). The Basque Country is a bilingual community in which Spanish and Basque coexist as official languages. All the children who participated in this study were fluent in both languages (early bilinguals) but received formal education in Basque only. None of the parents reported speaking any other language at home. Since this study was performed in Basque, children's language dominance was assessed through the expressive vocabulary section of the BEST (Basque English and Spanish Test), a test created specifically at BCBL for assessing participants' multilingualism (de Bruin, Carreiras, & Duñabeitia, 2017). The decision on the individual child's language dominance was supported by a short interview with the experimenter and by a subjective language questionnaire that the parents/tutors were asked to fulfil. The final language dominance measure was used to discard effects of this variable on the speech perception task. Information on the psycholinguistic profile of the children at the different testing times is provided below. Children from the two schools did not differ on their parents' or tutors' socioeconomic status defined based on their yearly net income (medium or medium‐high). Finally, no neurodevelopmental disorder (e.g. ADHD, ASD, etc.) was evident in any of the children. Parents reported children's normal hearing and normal or corrected‐to‐normal vision. None of the children had a family history of developmental language disorder and they were not at familial risk of any other cognitive‐related genetic pathology, as reported by the parents.

Children were tested under signed parental authorization at each of the testing times, and the whole longitudinal experiment was approved by the BCBL Ethics Review Board and complied with the guidelines of the Helsinki Declaration.

At t1, 32 children (age *M* = 5.11 years; *SD* = 0.29; 16 males) of the initial battery of 42 children who participated in the whole longitudinal study completed the speech perception task. The rest of the children (10) did not complete the task because of discomfort with the EEG cap. Twenty‐three and nine children were categorized as Basque or Spanish dominant respectively. Five of the children were left‐handers.

At t2, 34 (age *M* = 5.84 years; *SD* = 0.33; 17 males) out of the total sample of 38 children that came back for testing at t2 did the task. Twenty‐four of these children were Basque dominants and 10 Spanish dominants. Four children were left‐handers.

Finally, the 33 children that came back to complete the battery at t3 finished the task (age *M* = 6.98 years; *SD* = 0.32; 17 males). Twenty‐two and 11 children were characterized as Basque or Spanish dominant respectively. Four children were left‐handers.

### 2.2 Stimuli and procedure

Children were instructed to stare at a static child‐friendly image that appeared in the centre of the CTR screen while listening attentively through loudspeakers to Basque natural speech recorded by a native female speaker. Stimuli were delivered via loudspeakers at an 80 dB SPL with *Psychopy* (Peirce, 2009). For the recording of the text, the speaker was asked to read at a normal pace and not to over‐emphasize prosody (i.e. avoid infant directed speech). The children listened to a different unknown story over the three testing times to avoid attentional disturbances due to familiarity with the story. The recordings were six minutes long and were segmented in one‐minute fragments. After each of the six fragments, participants were asked a simple yes/no comprehension question (e.g. *Did the child eat the cake in the end?*) to ensure that they understood the story and were paying attention. These recesses were also used to allow the children to rest and move before resuming the experiment.

### 2.3 EEG data acquisition

The EEG signals were recorded in a child‐friendly soundproof electrically‐shielded room. Children were comfortably seated in a chair adapted to their size. They were instructed to stay silent and to avoid moving as much as possible. EEG signals were recorded using a Brain Products GmbH actiCAP with 32 electrodes. To reduce preparation time and avoid children's fatigue, we reduced the array of scalp electrodes to 19 (FP1, FP2, F7, F3, Fz, F8, F4, C3, Cz, C4, T7, T8, P7, P3, Pz, P8, P4, O1 and O2) which were distributed over the scalp based on 10‐10 International System. Additional reference electrodes were placed on both mastoids A1 and A2. In addition, we recorded electrode FCz and around the eyes at left Heog, right Heog and left Veog. Signals were sampled at 500 Hz with a high‐pass filter at 0.1 Hz. Monopolar differential recording was referenced online to electrode FCz.

### 2.4 Data analysis

#### 2.4.1 Data pre‐processing

The EEG data were pre‐processed and analysed in Matlab 2014b (Mathworks). After inspection of the raw data we decided to discard directly the data of four participants at t1 and of three participants at t2 due to excessive movement during recording. Unfortunately, the data of six of the children at t3 was not correctly recorded due to a technical problem and hence could not be used for further analysis. Consequently, the data of 28 (t1), 31 (t2) and 27 (t3) children were used in the pre‐processing stage. In each individual case, we inspected the signal visually and pooled channels with excessive noise from the signal of at least three surrounding electrodes (t1 *M*(*SD*) = 1.29(1.05); t2 *M*(*SD*) = 1.17(0.86); t3 *M*(*SD*) = 0.81(0.79)). Data were then bandpass‐filtered from 0.1 to 40 Hz with a Butterworth filter of order 4 applied twice: in the forward and then backward directions. The low pass cut‐off was set to 40 Hz, since no effects were expected at frequencies above that threshold (Bourguignon et al., 2013; Gross et al., 2013; Park, Ince, Schyns, Thut, & Gross, 2015). Further inspection of the data indicated the general presence of excessive noise in mastoid channels due to movements. Accordingly, we excluded electrodes A1 and A2 from further analysis, and re‐referenced the data offline to electrode Cz. This choice was based on previous works showing that this is an adequate reference electrode to examine hemispheric differences (Picton, 2011; Poelmans, Luts, Vandermosten, Ghesquière, & Wouters, 2012; Van der Reijden, Mens, & Snik, 2005; Van Dun, Wouters, & Moonen, 2009; Vanvooren, Poelmans, Hofmann, Ghesquiere, & Wouters, 2014). Next, we estimated 19 independent components from the data using fast ICA (Hyvarinen, 1999). Independent components corresponding to heartbeat, eyeblink and eye movements were identified and corrected (maximum of 2 components for each individual participant).

The audio signal was Hilbert‐transformed to obtain the broadband amplitude envelope of the audio signals (Drullman, Festen, & Plomp, 1994), band pass‐filtered from 0.1 to 40 Hz, and resampled time‐locked to EEG signals.

The pre‐processed signals (the audio stimuli and the EEG data) were segmented into 2,048‐ms‐long epochs with 1,024‐ms epoch overlap (Bortel & Sovka, 2007; Bourguignon et al., 2013; Molinaro et al., 2016). This epoch length led to a frequency resolution of ~0.5 Hz (inverse of the epoch duration), which is typical in speech‐to‐brain coherence analyses (Bourguignon et al., 2013; Molinaro et al., 2016). Epochs with amplitude more than 3 *z*‐values above the mean in at least one EEG channel were discarded. We did not further analyse datasets for which more than 30% of the data had to be rejected. This affected two children at t1, two children at t2 and one child at t3. Therefore, the data of 26, 29 and 26 participants were kept for t1, t2 and t3 respectively. For these data sets, an average percentage of 89.2% (*SD* = 11.03), 92.03% (*SD* = 2.99) and 94.13% (*SD* = 3.82) epochs was kept for the three testing times respectively, and submitted to the coherence analysis described hereafter.

#### 2.4.2 Coherence to natural speech

To calculate the relation (dependency) between the phase of EEG signals (*x*(*t*)) and the phase of speech signal (*y*(*t*)), we calculated *coherence* following the method proposed by Halliday et al. (1995). This method is a generalization of the Pearson correlation to the frequency domain. It quantifies the degree of coupling between two signals (here *x*(*t*) and *y*(*t*)) with a value between 0 and 1 (no linear and perfect linear relation respectively) for each frequency bin. Coherence was computed as follows:

$${\text{Coh}}_{\mathrm{xy}}\left(f\right)=\frac{{\left|P_{\mathrm{xy}}\left(f\right)\right|}^{2}}{P_{\mathrm{xx}}\left(f\right)P_{\mathrm{yy}}\left(f\right)},$$

the cross‐ and power‐ spectra of *x* and *y* are estimated as

$$P_{\mathrm{xx}}\left(f\right)=\frac{1}{N}\sum_{n=1}^{N}X_{n}\left(f\right)X_{n}^{*}\left(f\right),$$

$$P_{\mathrm{yy}}\left(f\right)=\frac{1}{N}\sum_{n=1}^{N}Y_{n}\left(f\right)Y_{n}^{*}\left(f\right),$$

$$P_{\mathrm{xy}}\left(f\right)=\frac{1}{N}\sum_{n=1}^{N}X_{n}\left(f\right)Y_{n}^{*}\left(f\right),$$

where *X_n_*(*f*) and *Y_n_*(*f*) denote the Fourier coefficients of the *n*th epoch of *x*(*t*) and *y*(*t*) at frequency *f*, and where *N* denotes the number of epochs available.

Coherence between the artifact‐free EEG data and the envelope of speech signal was calculated in the 0.1‐ to 40‐Hz frequency range, which yielded a coherence value for each possible combination of EEG channels, frequencies and subjects. Based on previous studies in adults showing significant coherence between the speech and the brain signals at ~0.5 Hz (Bourguignon et al., 2013; Clumeck et al., 2014) and at 4–8 Hz (Ding & Simon, 2012b; Peelle et al., 2013), we focused our analysis on these frequency ranges of interest. Note that coherence at the frequency bin corresponding to ~0.5 Hz actually reflects coupling in a range of frequencies *f* around 0.5 Hz, with a sensitivity profile proportional to the Fourier transform of a 2‐s‐long boxcar function: sinc((*f*–0.5 Hz)/0.5 Hz) (Destoky et al., 2019). Accordingly, coupling at the 0.5 Hz bin mainly reflects tracking in the 0.1–0.9 Hz range.

We assessed statistical significance at the level of the group with a non‐parametric permutation test (Nichols & Holmes, 2001). First, coherence was evaluated with the time‐flipped speech envelope signal. Coherence values with the genuine and flipped speech were averaged across subjects for each frequency range of interest. These values were then contrasted, providing a difference value for each electrode. We then computed the sample distribution of the maximum across electrodes of such contrast obtained after having randomly permuted genuine and flipped coherence values within subjects for a subset of 1,000 permutations. The 95th percentile of this distribution yielded a significance thresholds at *p* < .05 corrected for multiple comparisons for the initial contrast (Nichols & Holmes, 2001).

#### 2.4.3 Longitudinal trajectory of coherence to natural speech

Only channels that showed significant coherence to speech signals at any of the frequency ranges of interest in any of the testing phases were considered for the longitudinal analysis. In principle, since one of our main interests was to test the lateralization of coherence in children, we considered the possibility of averaging coherence values in the electrodes of the left and the right hemispheres. Nevertheless, to avoid obscuring intra‐hemispheric differences by averaging, the longitudinal trajectory of the separate electrodes was examined visually to detect differences among them. According to this, when the data suggested that the electrodes within the same hemisphere behaved differently along time, coherence values were not averaged per hemisphere, that is, they were entered separately in the analysis. Note that, in case of individual introduction of the electrodes, hemispheric information would already be coded implicitly in the Electrode factor, and in order to avoid losing degrees of freedom for parameter estimation, the Hemisphere factor was not entered in the model. In other words, we considered entering either the Electrode or the Hemisphere factor, but not both.

With the aim of evaluating statistically the progression of coherence in the electrodes of interest across the years, we fitted a mixed effects model using the *lme4* package available in R (Baayen, Davidson, & Bates, 2008; R Core Team, 2017). We chose this statistical test for two main reasons. First, the random effects by subject help to control for inter‐individual variability, reducing hence the weight of extreme observations for the calculation of group level statistics. Second, it has been recurrently suggested that this is the optimal currently available model fitting technique for longitudinal designs with missing data (e.g. Garcia & Marder, 2017; Laird & Ware, 1982).

Regarding the selection of the fixed effects, we used maximum likelihood (ML) estimation to fit several models and decide which fitted the data best. ML allows for the comparison of fits with identical random effects but different nested fixed effects (Zuur, Ieno, & Elphick, 2010). Specifically, we considered two different fixed effects structures that yielded two different models: an initial hypothesis‐driven model that included the factors Time (1, 2 and 3), Electrode/Hemisphere and their interaction (Time*Electrode/Hemisphere), and a second model that did not include the interaction (i.e. only Time and Electrode/Hemisphere factors). Model selection was then based on the comparison of the respective model's Akaike information criterion (AIC), maximum log likelihood units, on visual inspection of the coefficients (Gelman & Hill, 2006), and supported by our a priori hypotheses. Concerning the construction of the random effects, two by‐subject slopes for the effect of Time and Electrode/Hemisphere were entered to control for within‐subject variability and measures' interdependency. From there, the complexity of the random effects structure was increased in a series of models leading to a maximal model that converged and contained only the random effects that increased significantly the residual variance captured by the model (Barr, Levy, Scheepers, & Tily, 2013). Although parameters were estimated with ML for model comparison, the final model parameters were estimated with restricted maximum likelihood, since its estimation of the random effects improves when small sample sizes are involved as compared to ML (e.g. Morrell, 1998). We used the *R* package *lsmeans* (Lenth, 2016) to extract the model's least square means (LSMs; estimates), standard errors and confidence levels (CL) at 95% for each of the levels of the factors (and interactions) included in the fixed effects. Lastly, the *lsmeans* package was also used to explore post‐hoc contrasts. This package uses the Satterthwaite method to estimate *t*‐values, and their associated *p‐*values are corrected for multiple comparisons using the Tukey HSD method.

#### 2.4.4 Coherence and behavioural responses

Lastly, based on previous literature hinting at a close association between speech‐brain entrainment and intelligibility (e.g. Gross et al., 2013), we computed Pearson correlations between the coherence values in the different electrodes of interest and accuracy in the comprehension questions in order to examine if coherence was related to comprehension. In each of the analysis, children's IQ (Matrices score) and age in months was controlled for (i.e. partial correlations). False discovery rate (FDR) correction for multiple comparisons was calculated within each of the testing times.

## 3 RESULTS

### 3.1 Behavioural responses to the story

At t1, children answered correctly to a mean of 4.35 (*SD* = 1.38) of the six questions. The mean accuracy increased to 5.00 (*SD* = 0.96) at t2, and to 5.42 (*SD* = 0.76) at t3. Descriptively, these responses suggested that the speech was intelligible for the children, and that they were paying attention.

### 3.2 Coherence to natural speech

**Figure 1:**
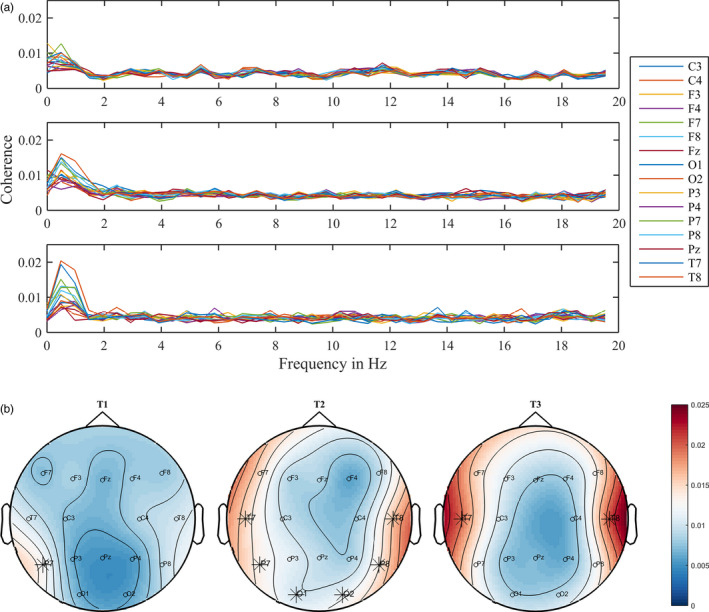
Developmental trajectory of coherence across the testing times. (a) Mean group coherence between 0 and 20 Hz across the electrodes in t1 (upper panel), t2 (middle panel) and t3 (bottom panel). The coloured lines represent the different electrodes. (b) Coherence maps at the 0.5 Hz frequency band across the testing times. Electrodes that showed significant coherence to speech are marked with an asterisk (*)

Group‐level speech‐brain entrainment between 0 and 20 Hz can be seen in Figure 1a. As shown in this figure, a clear peak in brain‐coherence to speech was visible only at delta frequencies (~0.5 Hz) at all testing times. Nevertheless, we further analysed the statistical significance of coherence in our second frequency range of interest: at theta frequencies (4–8 Hz).

#### 3.2.1 Coherence at t1

At the group level, the only channel that showed significant coherent activity to speech was P7. This electrode's signal was coherent with the audio signal at 0.5 Hz (low delta band; see upper panel of Figure 1a, and left plot of Figure 1b). No significant coherence was found at theta frequencies in any electrode.

#### 3.2.2 Coherence at t2

Again, we found significant mean group coherence to speech only at 0.5 Hz (and not in the 4–8 Hz range). Electrodes showing coherent activity were distributed along posterior right (T8, P8, O2) and posterior left (T7, P7) scalp locations (see middle panel of Figure 1a, and central plot of Figure 1b).

#### 3.2.3 Coherence at t3

As in t1 and t2, we found significant coherence to speech only at 0.5 Hz. This time, coherent activity was found in bilateral temporal electrodes (T7 and T8; see bottom panel of Figure 1a and right plot of Figure 1b).

### 3.3 Longitudinal trajectory of coherence to natural speech

**Figure 2:**
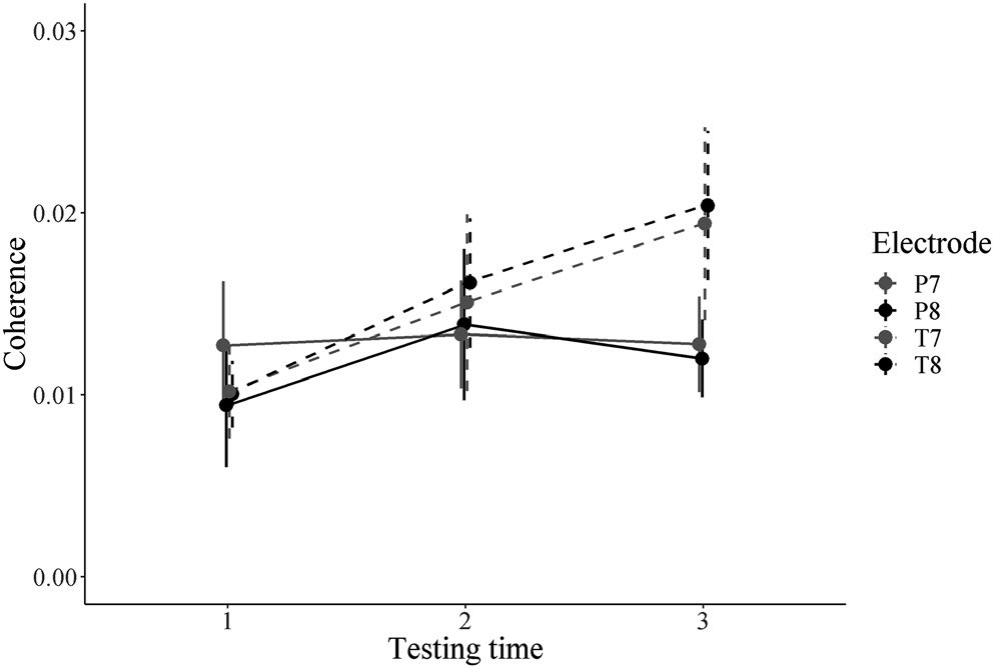
Development of coherence at 0.5 Hz in the electrodes of interest across the testing times. Vertical bars represent the standard error of the mean. Parietal electrodes are represented with solid lines and temporal electrodes, with dashed lines. Hemisphere is coded in the lines' colour (black: right; grey: left)

Based on the results of the coherence analysis, our only frequency range of interest was ~0.5 Hz. The electrodes considered for further analysis were T7, P7, T8, P8 and O2. Since significant coherence to natural speech has been shown mostly in temporal‐parietal sensors with MEG (Bourguignon et al., 2013; Gross et al., 2013; Molinaro et al., 2016; Peelle et al., 2013; Vander Ghinst et al., 2016), we decided to reduce our array of electrodes to T7, P7, T8 and P8 (electrodes of interest, from now on). Visual inspection of the longitudinal trajectory of the electrodes (see Figure 2) suggested that the trajectory of coherence values was different between intra‐hemispheric pairs (T7 vs. P7, T8 vs. P8). To avoid obscuring intra‐hemispheric differences by averaging activity within the hemispheres, we introduced the electrodes separately in our analysis (see Table 1 for descriptive statistics at the level of the group in the electrodes of interest).

**Table 1:** Mean group‐level coherence values (and standard deviation) at 0.5 Hz in the electrodes of interest. Electrodes that showed significant coherence are marked with an asterisk (*)

	Electrode											
	T7			P7			T8			P8		
TT	T1	T2	T3	T1	T2	T3	T1	T2	T3	T1	T2	T3
*M*	0.011	0.015*	0.019*	0.013*	0.013*	0.013	0.010	0.016*	0.020*	0.009	0.014*	0.012
*SD*	0.013	0.026	0.027	0.018	0.016	0.013	0.009	0.019	0.021	0.017	0.022	0.011

Abbreviation: TT, testing time.

#### 3.3.1 Model specification

The analysis of variance comparing the models with and without the interaction between our factors Time and Electrode indicated that the model that included the Time*Electrode interaction had a lower AIC (interaction model: −1,818.3; no interaction model: −1,821.5) and a larger log likelihood (interaction model: 943.13; no interaction model: 938.74), although the chi‐square test testing the statistical difference between the models did not reach significance (χ^2^(6) = 8.79; *p* = .19). Despite the latter and supported by our hypothesis that coherence values would change across the scalp in the different testing times, we chose the model with the interaction as the best fit of our data.

The final model included hence the fixed effects of Time (t1, t2 and t3), Electrode (T7, P7, T8 and P8) and their interaction, and the random by‐subject slopes for Time and Electrode initially entered in the model plus a random by‐subject intercept. The total number of different subjects included in the analysis amounted to 36, and the number of observations to 324.

#### 3.3.2 Model results

The summary of the model's random effects can be seen in Table 2a. The estimates of the model or LSMs (thereafter) together with their *SE* and upper and lower CL are presented in Table 2b. Inspection of the estimates suggested that the only electrodes whose coherence values increased along time were T7 and T8. Regarding the difference in coherence among the electrodes within each testing time, the only difference in the estimates that seemed relevant was within t3 and between parietal and temporal electrodes (P7 LSM: 0.12; P8 LSM: 0.11, T7 LSM: 0.18; T8 LSM: 0.20).

**Table 2:** Mixed effects model's output with the coherence value at 0.5 Hz as dependent variable and the Time, Electrode and Time*Electrode factors as fixed effects, and with by‐subject slopes for Time and Electrode and by‐subject intercept as random effects. (a) Output of the random effects structure. Reference levels are electrode P7 for the Electrode factor and T1 for the Time factor. (b) Fixed effects estimates (LSM), standard errors and confidence levels (0.95) for the different levels of the factors Time and Electrode

(a)	
Random effect	*SD*
Intercept	0.007
Electrode P8	0.005
Electrode T7	0.005
Electrode T8	0.005
T2	0.012
T3	0.006
Residual	0.010

(b)					
Time	Electrode	LSM	*SE*	Lower CL	Upper CL
1	P7	0.012	0.003	0.007	0.017
2		0.014	0.003	0.007	0.021
3		0.012	0.003	0.006	0.018
1	P8	0.008	0.003	0.003	0.014
2		0.014	0.004	0.006	0.022
3		0.011	0.003	0.005	0.017
1	T7	0.009	0.003	0.003	0.015
2		0.016	0.004	0.007	0.024
3		0.018	0.004	0.011	0.025
1	T8	0.009	0.002	0.004	0.014
2		0.016	0.004	0.009	0.024
3		0.020	0.003	0.014	0.026

We moved on to test statistically the significance of these effects by computing the pairwise contrasts with Tukey correction (from *R* package *lsmeans*). For parsimony reasons, only significant results will be discussed in text (complete results can be seen in Table 3). When the effect of Time on the different Electrodes was tested (see Table 3a), the only significant contrast was the one comparing t1 and t3 in Electrodes T7 and T8, meaning that there was an increase in coherence in these electrodes along time, while coherence did not increase or decrease in electrodes P7 or P8. Regarding the contrasts comparing the different electrodes within the testing times (see Table 3b), only the contrast between electrodes P8 and T8 within t3 was significant, such that coherence was stronger in the temporal as compared to the parietal electrode.

**Table 3:** Tukey‐corrected least square means (LSMs) contrasts. (a) Effect of Time on Electrode. (b) Effect of Electrode on Time

(a)				
Contrast (Time)	LSM	*SE*	*t*	*p*
Electrode P7				
1–2	−0.002	0.004	−0.49	0.88
1–3	−0.0001	0.003	−0.04	1.00
2–3	0.001	0.003	0.48	0.87
Electrode P8				
1–2	−0.006	0.004	−1.53	0.28
1–3	−0.003	0.003	−0.80	0.70
2–3	0.003	0.003	0.92	0.63
Electrode T7				
1–2	−0.007	0.004	−1.80	0.18
1–3	−0.01	0.003	−2.82	0.01*
2–3	0.003	0.003	−0.78	0.72
Electrode T8				
1–2	−0.007	0.004	−1.92	0.14
1–3	−0.01	0.003	−3.13	0.005*
2–3	−0.003	0.003	−0.92	0.63

(b)				
Contrast (Electrode)	LSM	*SE*	*t*	*p*
Time 1				
P7–P8	0.004	0.003	1.09	.69
P7–T7	0.003	0.003	0.97	.77
P7–T8	0.003	0.003	0.86	.82
P8–T7	−0.0004	0.003	−0.13	1.00
P8–T8	−0.0008	0.003	−0.25	.99
T7–T8	−0.0003	0.003	−0.1	1.00
Time 2				
P7–P8	−0.0004	0.003	−0.14	1.00
P7–T7	−0.002	0.003	−0.63	.92
P7–T8	−0.003	0.003	−0.86	.83
P8–T7	−0.002	0.003	−0.49	.96
P8–T8	−0.002	0.003	−0.75	.88
T7–T8	−0.0007	0.003	−0.23	1.00
Time 3				
P7–P8	0.001	0.003	0.32	.99
P7–T7	−0.006	0.003	−1.95	.21
P7–T8	−0.007	0.003	−2.30	.10
P8–T7	−0.007	0.003	−2.25	.12
P8–T8	−0.008	0.003	−2.72	.04*
T7–T8	−0.001	0.003	−0.35	.99

a Significant contrasts are marked with an asterisk (*).

### 3.4 Coherence and behavioural responses

Results of the partial correlations between delta‐coherence values in the electrodes of interest and accuracy in the comprehension questions can be seen in Table 4. As shown in this table, coherence values in electrode T8 correlated marginally with accuracy in response to the comprehension questions at t1, and significantly at t2 and t3. Note that, likely due to our modest sample size, none of these results was significant once FDR correction was applied.

**Table 4:** Partial correlations (controlling for IQ and age in months) between the 0.5 Hz coherence values in the electrodes of interest and accuracy in the responses to the comprehension questions

	Time	Electrode	Accuracy comprehension questions			
			*n*	*R*	*p*	FDR‐*p*
Coherence at 0.5 Hz	t1	P7	26	.16	.45	.52
		P8		.20	.33	.52
		T7		.13	.52	.52
		T8		.36	.06	.24
	t2	P7	29	.27	.14	.22
		P8		.24	.21	.22
		T7		.24	.22	.22
		T8		.43	.02*	.08
	t3	P7	26	−.02	.91	.91
		P8		.18	.38	.51
		T7		.23	.25	.50
		T8		.39	.05*	.20

a Significant correlations are marked with an asterisk (*).

## 4 DISCUSSION

This study examined the development of speech‐brain entrainment and its lateralization profile in young children. As expected, coherence to speech was found already when children were 4‐to‐5 years old (t1) and continued developing across the subsequent testing times (t2 and t3). Crucially, we found coherence to speech only at 0.5 Hz, replicating studies performed in adults with natural speech (Bourguignon et al., 2013; Gross et al., 2013; Molinaro et al., 2016). By contrast, our results did not hint at the presence of significant entrainment in the theta (4–8 Hz) range. This was somewhat surprising, given that theta entrainment has been consistently reported in previous works, including in some using the exact same coherence approach (Bourguignon et al., 2013, 2018; Molinaro et al., 2016; Vander Ghinst et al., 2016). This is however consistent with a recent MEG study in which speech entrainment was compared between adults and children aged 6–9 (Vander Ghinst et al., 2019). Based on 5‐min‐long recordings, theta entrainment was consistently found in adults, but only in half of the children, and with values barely exceeding statistical significance level. Given that 2–3 times more EEG than MEG data is needed to uncover significant speech entrainment (Destoky et al., 2019), it is not surprising that our EEG recordings of 1 min were not sufficient to identify these responses. In conclusion, we believe that the absence of the effect at theta frequencies could be rooted in technical (i.e. use of EEG) and/or in developmental reasons (i.e. in the language acquisition stage of our participants). The latter argument is also supported by previous behavioural results showing that children with immature linguistic and reading skills are also generally more sensitive to slow as opposed to fast information in the speech signal (Anthony & Francis, 2005; Ziegler & Goswami, 2005), which could explain why coherence was only found in the lowest (delta) frequencies.

### 4.1 Developmental trajectory of delta‐band coherence to speech

Our results revealed interesting effects on the developmental trajectory of delta speech‐brain entrainment during early childhood. Although our phase‐by‐phase coherence analysis – which was performed in order to reduce the array of electrodes of interest – seemed to reveal that coherence was only present in the left hemisphere at t1, the statistical comparison across testing times and hemispheres did not reveal significant inter‐hemispheric differences in coherence to speech in temporal or parietal sites of the scalp at any testing time. Based on these results, we could conclude that, at least with our stimulus and sample, the right‐hemispheric bias found for delta speech‐brain entrainment in older children and adults in auditory areas (e.g. Bourguignon et al., 2013; Gross et al., 2013; Lizarazu et al., 2015; Molinaro & Lizarazu, 2018; Molinaro et al., 2016) has not yet been attained at around 6–7 years of age. The fact that we also found significant delta‐coherence to speech in the left hemisphere is at odds with previous hemodynamic evidence in infants that found right‐lateralized responses to speech prosody (Homae et al., 2006; Perani et al., 2011; Telkemeyer et al., 2009, 2011). By contrast, our results are in line with infants' studies reporting no hemispheric differences in the oscillatory responses to speech and to speech‐relevant amplitude modulations (Peña et al., 2010; Telkemeyer et al., 2011). In addition, the presence of significant delta coherence in the left hemisphere during early childhood is supported by a previous study with 7‐month‐old children which found phase coherence to low‐pass filtered speech to be left‐lateralized (Kalashnikova et al., 2018) and by a different study from our lab which also found left‐lateralized coherence to speech in 5‐year‐old children (Pérez‐Navarro, Molinaro, & Lallier, 2018, poster communication).

### 4.2 Relation between delta coherence and linguistic experience

Importantly, the logical progression of the coherence phenomenon and specially the increase across phases in temporal electrodes suggests that our results are not due to global developmental changes in electrophysiological activity (Benninger, Matthis, & Scheffner, 1984; Thatcher, 1992), but are rather specific to our linguistic stimulus. Indeed, the only statistically significant increase in coherence across the testing times was found between t1 and t3 and in bilateral temporal electrodes (T7 and T8). Because of the auditory nature of our stimulus, the fact that coherence increased significantly only in temporal sites (i.e. adult‐like topography at the sensor level; Bourguignon et al., 2013; Gross et al., 2013; Molinaro et al., 2016) supports the idea that the effect was related to the interaction between age and the linguistic stimulus, and not merely to electrophysiological changes related with age.

Contrary to our expectations, we did not find reading acquisition to influence hemispheric specialization for processing the slow temporal components of speech, a hypothesis that was based on the corpus of evidence showing that interhemispheric differences in favour of the right hemisphere are positively correlated with reading achievement in older children (Abrams et al., 2009; Lizarazu et al., 2015; Molinaro et al., 2016). Here it is important to note that, at t3, the children of the present study had only received 1 year of formal reading instruction (i.e. were beginning readers) and we cannot discard that lateralization changes occur later in the reading acquisition process, that is, once reading is more automatized. To test this hypothesis, and to establish a conclusive relation between literacy acquisition and speech‐brain entrainment development, it would be necessary to follow‐up the children until later stages, and crucially to also measure reading abilities. We hope that future studies will contribute to shed light on this doubtless thrilling question, whereas our results only allow us to state that, at least with our sample and methods, delta coherence to speech seems to be bilateral at 7 years of age.

On its own, our result that the delta‐band oscillatory response to the slow temporal components of speech is not significantly different between hemispheres in young children could be supported by at least two accounts outside the reading literature. First, a previous study in the field of syntax has shown that 6‐years old children's lateralization pattern for language processing is different from the one in adults, such that inter‐hemispheric connections prevail in the child brain, whereas the adult brain shows a highly specialized left‐hemisphere network for language processing (Friederici, Brauer, & Lohmann, 2011). In line with this evidence, our results suggest that hemispheric specialization for speech perception is still not in place at 7 years of age. Second, we cannot discard that the psycholinguistic profile of our participants had some influence in the bilateral response pattern we report. Our participants were Basque‐Spanish bilinguals from birth or learnt both languages before their third year of life, and early bilinguals have been shown to process both native languages in a more bilateral manner, as opposed to late bilinguals and monolinguals, for which native language processing is normally left‐lateralized (Hull & Vaid, 2007). In conclusion, our and previous results might be influenced by the task, the age and the neuropsychological profile of the participants tested, and hence considering these variables seems necessary to describe, predict, and explain the astonishing complexity of language development during childhood.

Lastly, it is important to highlight that the evidence on the development of hemispheric lateralization for language operations is controversial in general. On the one hand, positive results on a bilateral‐to‐lateralized trajectory of activation from childhood into adulthood has been reported using neurophysiological (Kadis et al., 2011; Pihko et al., 2005; Ressel et al., 2008; Spironelli & Angrilli, 2009) and hemodynamic (Brown et al., 2005; Holland et al., 2001; Szaflarski et al., 2006) measures. On the other hand, several studies failed to find lateralization differences across age groups (Gaillard et al., 2003; Papanicolaou et al., 2006; Wood et al., 2004). The development of hemispheric specialization for different linguistic aspects is hence a complex issue that demands further scientific work, and ideally studies which take into account the linguistic experiences that children undergo at the specific testing time.

### 4.3 Delta coherence and intelligibility

Our data provide preliminary evidence that delta coherence in the right hemisphere could be related to intelligibility, as previously shown in adults (Gross et al., 2013; Molinaro & Lizarazu, 2018). Accuracy in the responses to the comprehension questions was associated positively with delta‐coherence values in electrode T8 only. Although, due to the age of the children, accuracy in this simple and indirect behavioural measure could also be related to other cognitive variables such as attention to the speech stream, our results provide preliminary evidence that delta coherence to speech in the right hemisphere might be related to intelligibility since early childhood. Please note that these analyses involved too large a number of comparisons for our sample size, and results did not survive FDR correction. Future studies with larger sample sizes must be carried out in order to draw definite conclusions on the relation between right‐hemispheric delta coherence and intelligibility in young children.

### 4.4 Limitations and future directions

It is important to note that, even though the coherence values were significant in temporo‐parietal sites of the scalp after correction for multiple comparisons was applied, the coherence values of the children were generally low even in our last testing time (range from 0.0004 to 0.12). Nevertheless, in the field of physiology, coherence values below 0.10 have been consistently reported across studies examining the relation between brain and electromyographic activity, among others (Bourguignon et al., 2013; Conway et al., 1995; Gross et al., 2005; Pohja, Salenius, & Hari, 2005; Pollok, Gross, Dirks, Timmermann, & Schnitzler, 2004; Salenius, Portin, Kajola, Salmelin, & Hari, 1997).

Ideally, in order to make more solid claims on the developmental trajectory of speech‐brain entrainment across the life‐span, it would be optimal either to follow up participants along a longer period of time (i.e. until they are adults) or to directly compare different age groups (i.e. cross‐sectional designs), and ideally both (i.e. sequential designs). We opted for a longitudinal design in very young children because of several reasons. First of all, the enterprise of measuring coherence to natural speech longitudinally in children was never undertaken before in the literature. Furthermore, the task of finding linguistic stimuli that are equally engaging both for adults and children samples is still unresolved, which leaves open the possibility that differences between age groups are not intrinsically related to the phenomenon that we intend to study (e.g. differences in engagement, attention, etc.). Finally, a recent study comparing adults and children (Vander Ghinst et al., 2019) demonstrated that the level of theta entrainment was drastically lower in children (aged 6–10) than in adults. Specifically, theta entrainment was seen in only half of the kids based on 5‐min of MEG recording. Related to this, it has been showed that two‐ or three‐times longer recordings are needed in EEG as compared to MEG in order to estimate speech entrainment (Destoky et al., 2019). We believe that these pieces of evidence clearly suggest that it was normal that we did not find significant entrainment at theta frequencies in our group of children, and that we would undoubtedly find it in adults. In sum, the above substantially diminishes the potential added value of including group of adults in our study. In any case, we hope that our pioneering work catalyses future studies with optimized designs and acknowledge that there is still a long way to go in order to characterize the trajectory of the speech‐brain entrainment phenomenon along the life cycle.

As mentioned earlier, our sample was made of bilingual children. In order to discard that our EEG results were associated with children's language of dominance (Basque vs. Spanish), we carried out a follow‐up analysis. Results of pair‐wise Spearman correlations showed that coherence values did not correlate significantly with the Language dominance measure (all *p*s > .20). In any case, we cannot discard the possibility that our findings on speech‐brain entrainment are partially due to the children's psycholinguistic profile. We hope that future studies using more sophisticated measures to assess the children's degree of bilingualism, or studies comparing directly groups of monolingual and bilingual children, will be able to answer this question.

Since brain coherence had never been evaluated in such young children and the nature of the study was hence mostly exploratory, we settled for a rather small sample size as a first approach to the field in the hope that future studies with more statistical power will further address the questions explored in the current work.

Lastly, coherence measures also have limitations. Coherence provides one single value across the entire time course of the experiment. Alternative measures that provide time‐resolved estimates such as TRF (Crosse, Di Liberto, Bednar, & Lalor, 2016) could have been pursued, although the ability of these measures to evaluate entrainment has been recently questioned (Doelling et al., 2019). We hope that future studies pursuing optimized time‐resolved analyses will provide more information on the dynamics of the brain response to speech.

## 5 CONCLUSIONS

This work corroborates that the speech‐brain entrainment phenomenon at low (delta) frequencies occurs already in early childhood, which supports a relevant biological meaning of this phenomenon for the establishment of adult‐like speech perception abilities. Moreover this is the first study to provide data on its longitudinal trajectory. Coherence to speech increased significantly only in temporal electrodes along the testing times. The fact that we found no inter‐hemispheric differences in the children's oscillatory responses suggests that at 7 years of age, typical right‐hemispheric specialization for speech delta‐frequency components is not in place. Lastly, delta speech‐brain coherence was positively associated with an indirect measure of intelligibility, suggesting that the entrainment phenomenon might support core linguistic operations since early childhood.aaa

## CONFLICT OF INTEREST

The authors declare that the research was conducted in the absence of any commercial or financial relationships that could be construed as a potential conflict of interest.

## Data Availability

The data that support the findings of this study are available from the corresponding author upon reasonable request.
